# Prokineticin 2 expression as a novel prognostic biomarker for human colorectal cancer

**DOI:** 10.18632/oncotarget.25706

**Published:** 2018-07-10

**Authors:** Yu Yoshida, Takanori Goi, Hidetaka Kurebayashi, Mitsuhiro Morikawa, Yasuo Hirono, Kanji Katayama

**Affiliations:** ^1^ First Department of Surgery, University of Fukui, Fukui, Japan; ^2^ Cancer Care Promotion Center, University of Fukui, Fukui, Japan

**Keywords:** colorectal cancer, prokineticin 2, molecular biomarker, prognostic factor, liver recurrence

## Abstract

Molecular tumor biomarkers hold considerable promise for accurately predicting colorectal cancer (CRC) recurrence and progression. Prokineticin 2 (PROK2) may be associated with angiogenesis and tumor formation in some malignant tumors. However, its prognostic value remains unknown. We focused on the association between PROK2 expression and clinical characteristics of CRC to assess value of PROK2 as a potential biomarker for stage I–III CRC patients prognosis.

Between 1992 and 2006, 436 consecutive patients with stage I–III CRC treated with curative resection were included. PROK2 expression in primary tumors was investigated using immunohistochemistry. An animal model of liver metastasis was used to assess the role of PROK2.

Positive PROK2 expression in primary tumors was found in 222 of 436 (50.9%) human CRC specimens and was significantly associated with lymphatic invasion, lymph node metastasis, clinical stage, and postoperative liver recurrence rate. Recurrence-free survival was significantly shorter in patients with positive PROK2 expression than in those with negative PROK2 expression. PROK2 expression was an independent unfavorable prognostic indicator for CRC [hazards ratio, 2.119; 95% confidence interval, 1.315–3.415; p = 0.002]. PROK2 overexpression promoted liver metastasis *in vivo*.

We suggest that positive PROK2 expression is observed in CRC primary tissues; thus, PROK2 may be a useful predictor for liver recurrence and prognosis in CRC.

## INTRODUCTION

Colorectal cancer (CRC) is a significant health problem, representing the third most common cancer worldwide [1–3]. In Japan, 124,921 new cases of CRC were reported in 2011, and 48,485 deaths from CRC were reported in 2014 [4]. Despite major advances in surgical techniques and devices and treatment modalities, such as chemotherapy, immunotherapy, and radiotherapy, the prognosis of patients with CRC remains poor because of distant metastasis and recurrence [5, 6]. Generally, the identification of patients who were at high risk of recurrence depended on pathological characteristics, such as the depth of invasion, nodal metastasis, stage group, and perforation or invasion of adjacent organs [7]. Nevertheless, the clinical outcomes are diverse among patients, including those with similar clinicopathological parameters and treatments, because CRC is a biologically heterogenous disease and causes dysfunction of multiple proteins that control cell proliferation and survival [8–10]. Patients who are at the same clinical stage of CRC might have distinct molecular drivers and different prognoses. Thus, there is an urgent need to identify appropriate biomarkers to improve the prognosis prediction and clinical outcomes among patients with CRC.

Prokineticin 2 (PROK2) is a cysteine-rich secreted protein that is expressed in the testis and, at lower levels, in the small intestine. It is a part of the prokineticin protein family, which has a conserved N-terminal sequence of AVITGA and 10 cysteines. The gene encoding PROK2 is located on chromosome 3p21.1 and is associated with Kallmann syndrome and hypogonadotropic hypogonadism with normal olfactory function [11–13]. Recent studies have proposed that PROK2 participates in numerous essential physiological processes, including inflammation, neurogenesis, tissue development, angiogenesis, regulation of the circadian clock, and nociception [14–16]. Regarding malignant tumors, some reports have shown that PROK2 is related to angiogenesis in glioblastomas [17] and hepatocellular carcinomas [18], cell infiltration in pancreatic cancer [17, 19], and tumor development in prostate cancer [20]. These findings highlight the relationship of PROK2 with malignant tumors. Our previous study found that PROK2 expression is associated with angiogenesis and tumor formation in CRC [21]. However, the prognostic value of PROK2 expression in human CRC remains unclear.

In this study, we investigated PROK2 expression using immunostained specimens from patients with CRC who were treated with curative resection. Additionally, we evaluated the association between PROK2 expression and its clinical significance to assess the value of PROK2 as a potential biomarker for the prognosis of patients with stage I–III CRC. Furthermore, to evaluate the effects of PROK2 on CRC metastasis, we assessed whether PROK2 overexpression promotes liver metastasis in a mouse model.

## RESULTS

### PROK2 expression in human CRC

**Figure 1:**
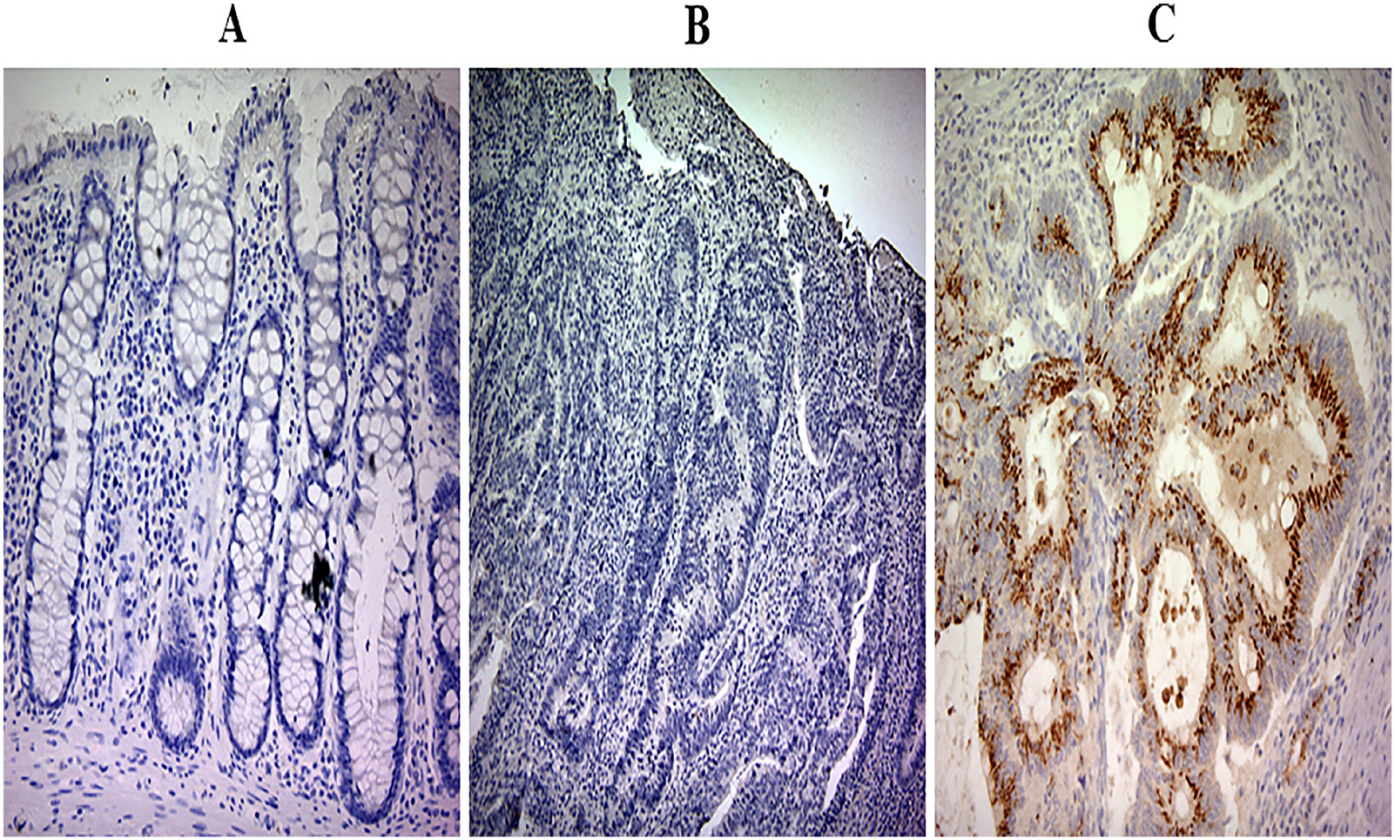
Prokineticin 2 (PROK2) protein expression in colorectal cancer (CRC) tumor tissues and adjacent normal tissues **(A)** Representative image showing immunohistochemical analysis of PROK2 expression in adjacent normal tissue. Magnification, ×100. In all normal colonic epithelial samples, PROK2 protein immunoreactivity is absent. **(B)** Negative expression of PROK2 protein in CRC tissue. Magnification, ×100. **(C)** Positive expression of PROK2 protein in CRC tissue. Magnification, ×100. The PROK2 protein is strongly expressed and localized in the cytoplasm.

Figure 1 shows exemplary cases of CRC. PROK2 immunostaining positivity was observed as yellow–brown granules in the cytoplasm but not in the nucleus (Figures 1B and 1C). Positive immunoreactivity of protein PROK2 was detected in 222 (50.9%) of 436 primary CRC tissue samples on semiquantitative analysis. However, in normal colorectal mucosa among the 436 samples, the immunoreactivity of protein PROK2 was clearly absent in both the cytoplasm and nucleus (Figure 1A).

### Correlations of PROK2 expression with clinicopathological parameters

There were no significant associations between PROK2 expression and the following clinicopathological factors: sex, age, tumor location, tumor sideness, histological type, venous invasion, and serosal invasion. On the other hand, PROK2 expression was significantly correlated with lymphatic invasion and lymph node metastasis. Furthermore, PROK2 expression was positively correlated with tumor-node-metastasis (TNM) stage, and patients with an advanced TNM stage showed higher PROK2 expression (Table 1).

**Table 1:** Correlation between prokineticin 2 (PROK2) expression in primary tumors and clinicopathological characteristics of patients with stage I–III colorectal cancer

	PROK2 positive		
	Total No of cases	No of cases (%)	P-value
All cases	436	222 (50.9)	
Gender			0.073
Male	248	117 (47.2)	
Female	188	105 (55.9)	
Age (average, 69.4 years)			0.880
<65 years	138	71 (51.4)	
≥65 years	298	151 (50.7)	
Tumor location			0.289
Colon	329	162 (49.2)	
Rectum	107	60 (56.1)	
Tumor sideness			0.996
Right side	161	82 (50.9)	
Left side	275	140 (50.9)	
Histological type			0.788
WD + MD	407	209 (51.4)	
PD	13	6(46.2)	
Mucinous	16	7 (43.8)	
Lymphatic invasion			0.002
Negative	210	91 (43.3)	
Positive	226	131 (58.0)	
Venous invasion			0.643
Negative	137	72 (52.6)	
Positive	299	150 (50.2)	
Serosal invasion			0.435
Negative	210	111 (52.9)	
Positive	226	111 (49.1)	
N (TNM 6th)			0.001
N0	259	106 (40.9)	
N1, N2	177	116 (65.5)	
Stage (TNM 6th)			0.001
I	92	38 (41.3)	
II	168	71 (42.3)	
III	176	113 (64.2)	

WD: well differentiated, MD: moderately differentiated, PD: poorly differentiated.

### Relationship between PROK2 expression and the liver recurrence rate based on CRC stage

As shown in Table 2, liver recurrence was noted in 73 (16.7%) patients. PROK2 expression indicated high liver recurrence risk in patients with localized CRC, particularly those with stages II and III CRC. Among patients with stage II and III CRC, the liver recurrence rate was significantly higher in those who showed PROK2 expression in the primary tumor than in those who did not show PROK2 expression (stage II: 21.1% vs. 8.2%, p = 0.016; stage III: 33.6% vs. 21.4%, p = 0.022). Among patients with stage I CRC, no significant difference in the liver recurrence rate was noted between those who showed PROK2 expression in the primary tumor and those who did not show PROK2 expression.

**Table 2:** Correlation between prokineticin 2 (PROK2) expression and the recurrence rate of liver metastasis based on the individual colorectal cancer stage

Stage Grouping	PROK2 negative			PROK2 positive			P-value
	No of cases	Recurrence	%	No of cases	Recurrence	%	
All cases	214	19	8.9	222	54	24.3	0.001
I	55	0	0	38	1	2.6	0.226
II	97	8	8.2	71	15	21.1	0.016
III	70	15	21.4	113	38	33.6	0.022
II and III	160	19	11.9	184	53	28.8	0.001

### Association between PROK2 expression and recurrence-free survival (RFS) rate

**Figure 2:**
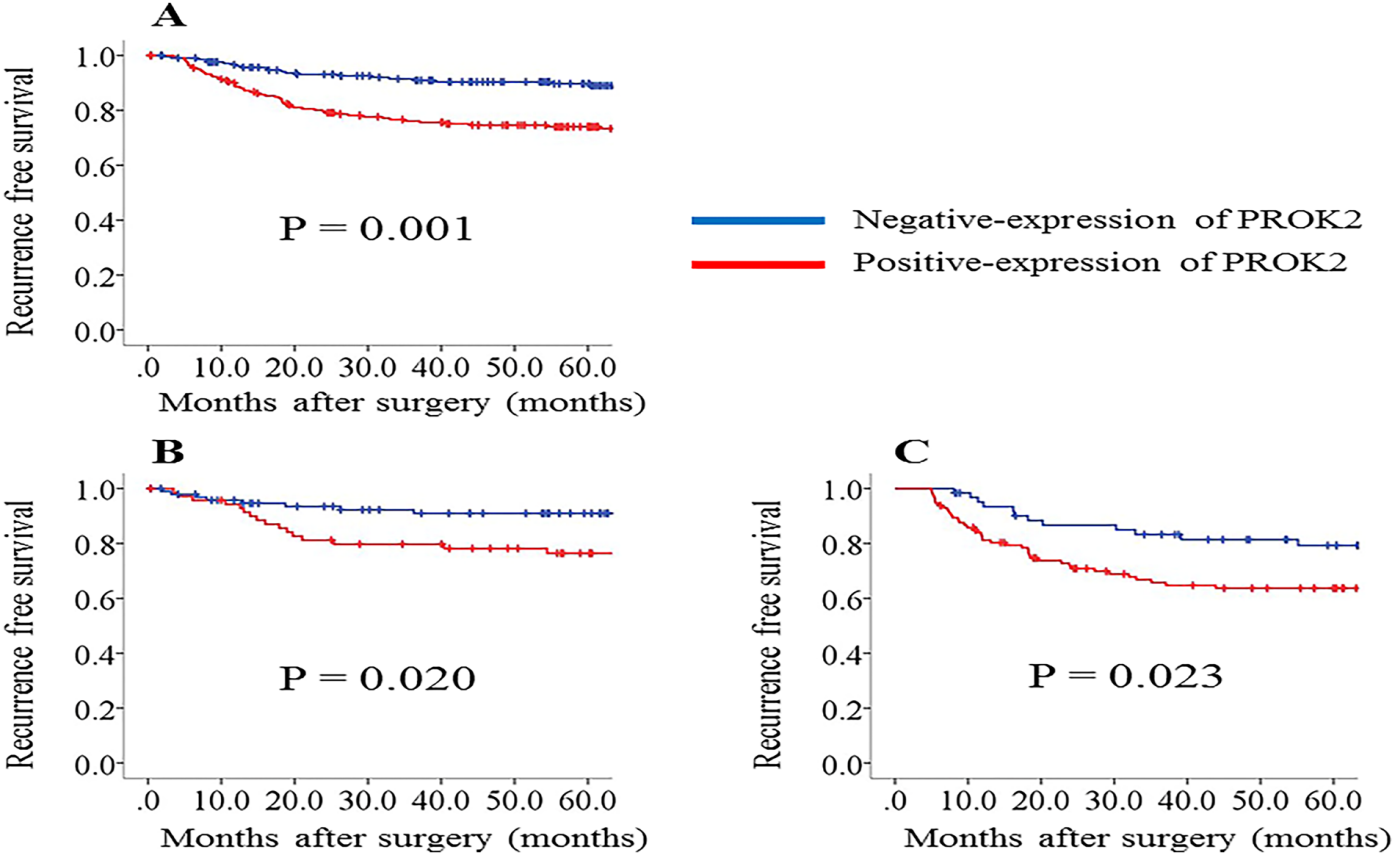
Recurrence-free survival (RFS) rates compared between positive and negative prokineticin 2 (PROK2) expression **(A)** All cases (n = 436). **(B)** Stage II cases (n = 168). **(C)** Stage III cases (n = 176). RFS is significantly poorer in the positive PROK2 expression group than in the negative PROK2 expression group in all cases as well as in individual-stage cases.

Among all the 436 patients, the 5-year RFS rate was 87.0% for patients with PROK2-negative primary tumors and 78.6% for patients with PROK2-positive tumors (p = 0.001; Figure 2A). With regard to the tumor stage, we found that RFS duration was significantly shorter in patients with PROK2-positive tumors than in those with PROK2-negative tumors among patients with stage II (p = 0.020; Figure 2B) and III CRC (p = 0.023; Figure 2C). However, among patients with stage I CRC, there was no significant difference in the RFS duration between patients with PROK2-positive and -negative tumors. To determine whether positive PROK2 expression in the primary tumor could serve as an independent prognostic marker for RFS in patients with CRC, we performed univariate and multivariate analyses. The univariate analysis showed that poor RFS in patients with CRC was associated with positive PROK2 expression (p = 0.001), histological type (p = 0.002), lymphatic invasion (p = 0.043), venous invasion (p = 0.043), and lymph node metastasis (p = 0.001) (Table 3). The multivariate analysis of these factors revealed that positive PROK2 expression [hazards ratio (HR), 2.119; 95% confidence interval (CI), 1.315–3.415; p = 0.002], histological type (HR, 2.688; 95% CI, 1.425–4.995; p = 0.002), and lymph node metastasis (HR, 2.311; 95% CI, 1.149–3.764; p = 0.001) were significant independent prognostic indicators for RFS in patients with CRC (Table 3).

**Table 3:** Univariate and multivariate Cox analyses for recurrence-free survival (RFS) in a Cox proportional hazard model

Factors	Univariate analysis			Multivariate analysis		
	HR	95% CI	P-value	HR	95% CI	P-value
PROK2 (Positive vs Negative)	2.409	1.520–3.817	0.001	2.119	1.315–3.415	0.002
Gender (Male vs Female)	0.879	0.706–1.093	0.246			
Age (<65 vs ≥65 years)	0.816	0.658–1.012	0.064			
Tumor location (Colon vs Rectum)	0.896	0.664–1.208	0.471			
Tumor sideness (Right vs Left)	1.138	0.727–1.779	0.572			
Histological type (WD/MD vs PD/Muc)	2.683	1.457–4.942	0.001	2.688	1.425–4.995	0.002
Serosal invasion (Positive vs Negative)	1.233	0.806–1.888	0.335			
Lymphatic invasion (Positive vs Negative)	1.566	1.015–2.414	0.043	1.009	0.638–1.597	0.969
Venous invasion (Positive vs Negative)	1.677	1.016–2.766	0.043	1.417	0.843–2.381	0.189
Lymph node metastasis (Positive vs Negative)	3.057	1.961–4.766	0.001	2.311	1.149–3.764	0.001

HR: hazard ratio, CI: confidence interval, WD: well differentiated, MD: moderately differentiated, PD: poorly differentiated, Muc: mucinous.

### PROK2 expression in CRC cell lines

**Figure 3:**
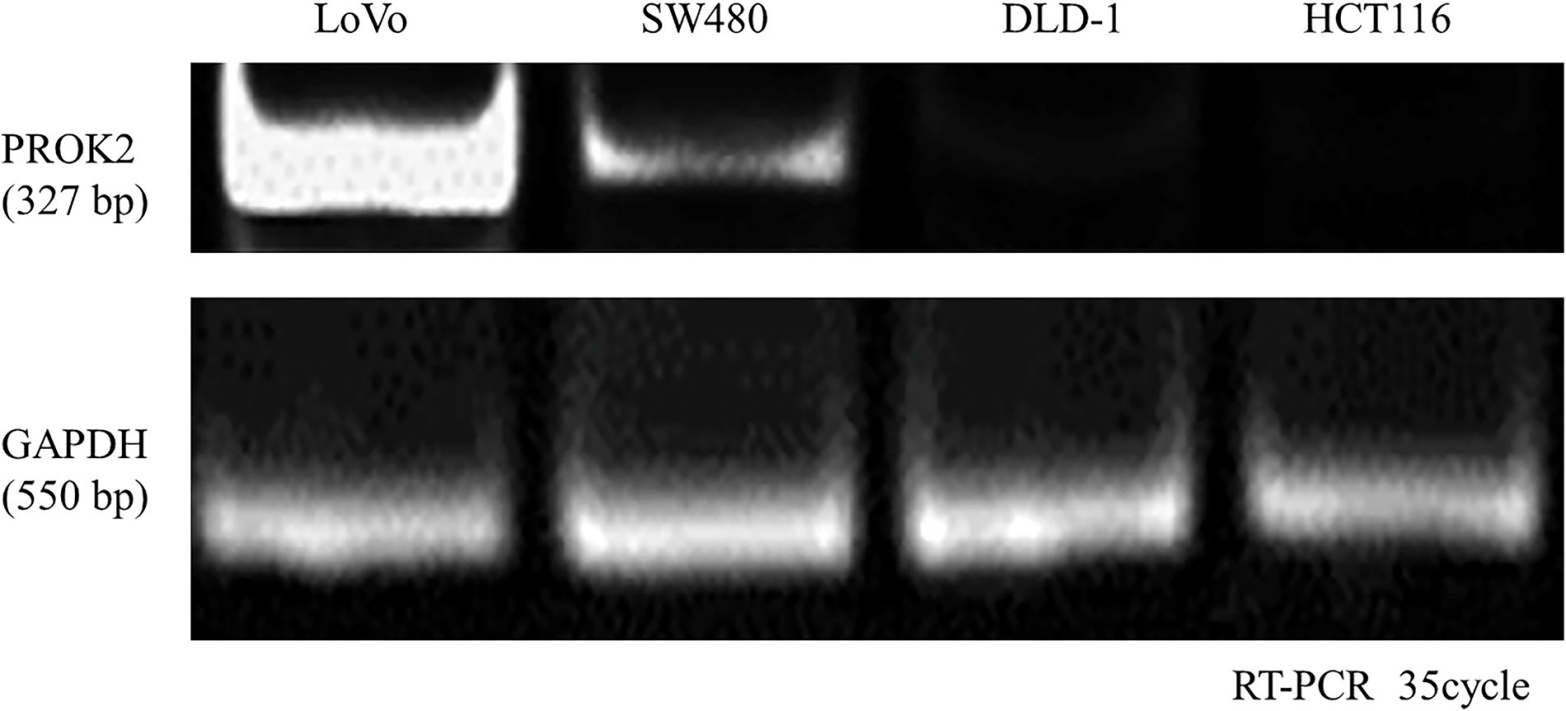
Prokineticin 2 (PROK2) expression in human colorectal cancer (CRC) cell lines (LoVo, SW480, HCT116, and DLD-1) PROK2 mRNA expression analyzed by reverse transcription-polymerase chain reaction. Although PROK2 mRNA expression is not observed in HCT116 and DLD-1, it is observed in LoVo and SW480.

We evaluated PROK2 mRNA expression in four CRC cell lines (LoVo, SW480, DLD-1, and HCT116) using reverse transcription-polymerase chain reaction (RT-PCR). All the values were normalized to those of glyceraldehyde-3-phosphate dehydrogenase (GAPDH). PROK2 mRNA expression was detected in LoVo and SW480 cell lines. PROK2 mRNA was assessed using band sequencing (Figure 3).

### Transfection of PROK2 into CRC cell lines with low PROK2 mRNA expression

**Figure 4:**
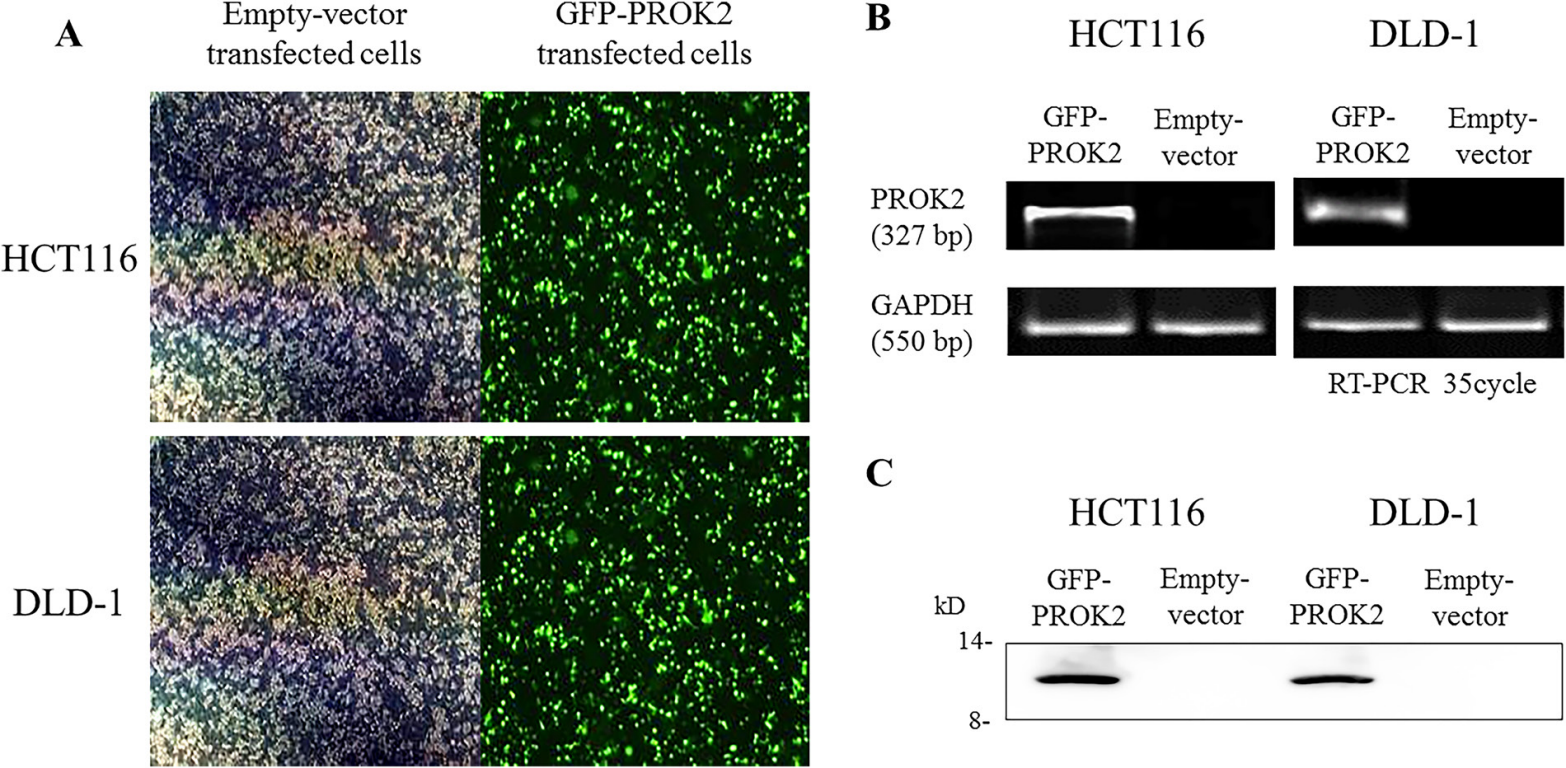
The identification of Prokineticin 2 (PROK2) overexpression in human colorectal cancer (CRC) cell lines (HCT116 and DLD-1) **(A)** PROK2 overexpression in CRC cells (fluorescence microscopy). CRC cells were transfected to overexpress PROK2 vector or empty vector alone. The cells expressing the protein were assessed using focal laser microscopy. **(B)** Overexpression of PROK2 mRNA was investigated using RT-PCR in HCT116 (left panel) and DLD-1 (right panel) cell lines. **(C)** Overexpression of the PROK2 protein was examined using western blotting with anti-PROK2 antibody in HCT116 (left panel) and DLD-1 (right panel) cell lines.

The pCMV6-Ac-GFP-PROK2 vector was transfected into CRC cell lines having low PROK2 mRNA expression (DLD-1 and HCT116). PROK2 mRNA overexpression was identified using fluorescence microscopy (Figure 4A) and RT-PCR (Figure 4B). Furthermore, western blot analysis demonstrated PROK2 protein overexpression in PROK2-transfected HCT116 and DLD-1 compared with pCMV6-Ac-empty vector-transfected cells (Figure 4C).

### PROK2 promoted liver metastasis of CRC cell lines *in vivo*

**Figure 5:**
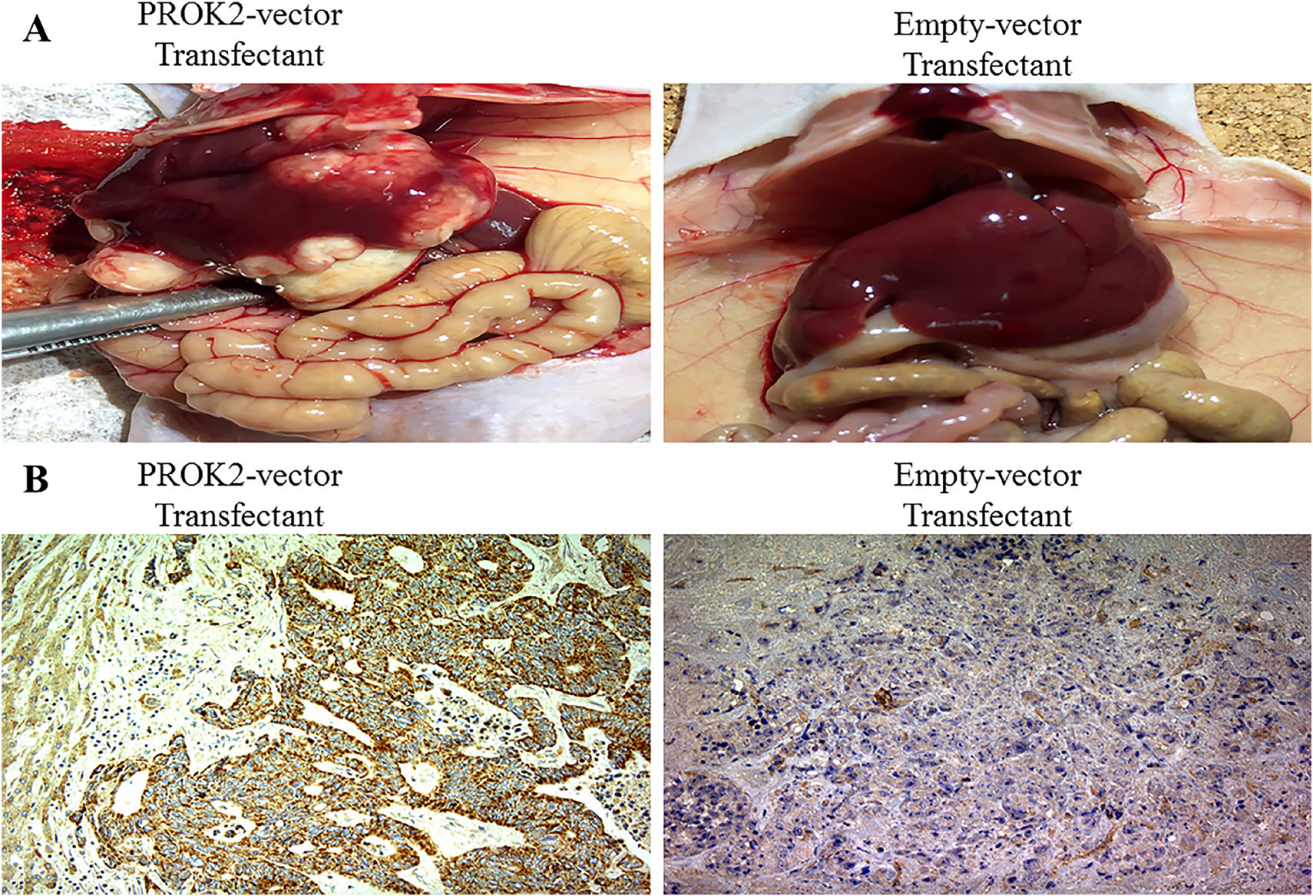
A nude mouse tumor xenograft model **(A)** Representative images of the livers of nude mice injected with PROK2-transfected DLD-1 cells vs. empty vector-transfected DLD-1 cells. Left image is a typical view of the liver presenting with macroscopic metastasis. **(B)** Histologic evaluation of mouse metastatic liver nodules injected with PROK2-transfected DLD-1 cells (left image; magnification, ×100) vs. empty vector-transfected DLD-1 cells (right image; magnification, ×100) using immunohistochemical analysis with the anti-PROK2 antibody.

**Figure 6:**
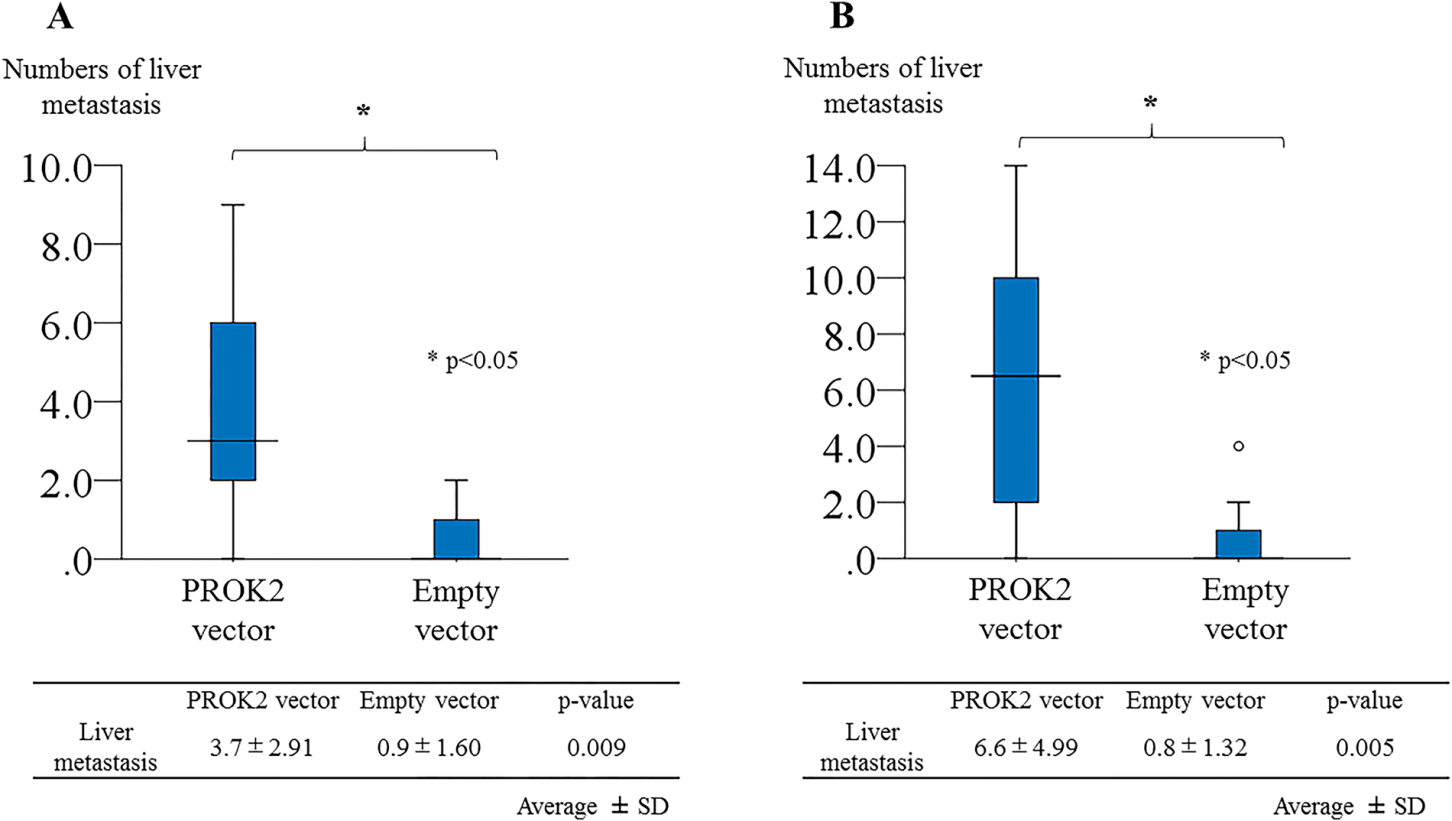
Prokineticin 2 (PROK2) promoted liver metastasis after splenic injection of colorectal cancer cells *in vivo* **(A)** Number of liver metastasis nodules with PROK2-transfected HCT116 vs. empty vector-transfected HCT116 cells (n = 20). **(B)** Number of liver metastasis nodules with PROK2-transfected DLD-1 vs. empty vector-transfected DLD-1 cells (n = 20). ^*^p < 0.05 vs. empty vector-transfected cells.

To detect the effects of PROK2 on the metastatic ability of CRC cells, a nude mouse tumor xenograft model was constructed. Four weeks after splenic injection of empty vector-transfected HCT116/DLD-1 or PROK2-transfected HCT116/DLD-1, macroscopic and microscopic metastases to other organs were evaluated (Figure 5A). In immunohistochemical analysis with the anti-PROK2 antibody (Novus Biochemicals) of liver metastatic nodules, we observed positive staining of PROK2 protein in liver metastases derived from PROK2-transfected HCT116/DLD-1. In contrast, PROK2 protein was not detected in liver metastases derived from empty vector-transfected HCT116/DLD-1 (Figure 5B). Four weeks after implantation, the mean number of liver metastases was 3.7 ± 2.91 in PROK2-transfected HCT116 and 0.9 ± 1.60 in empty vector-transfected HCT116 (p = 0.009; Figure 6A). In addition, the mean number of liver metastases was 6.8 ± 4.99 in PROK2-transfected DLD-1 and 0.8 ± 1.32 in empty vector-transfected DLD-1 (p = 0.005; Figure 6B). Our results showed that PROK2 transfection promoted liver metastasis of CRC cells and significantly increased the mean number of liver metastatic nodules. These results confirmed that PROK2 accelerated liver metastasis of CRC cells *in vivo*.

## DISCUSSION

CRC is one of the most common malignancies worldwide and is a major cause of cancer-related mortality [1-3, 22]. Despite improvements in CRC treatments, approximately 20%–50% of patients treated with curative surgery subsequently experience disease recurrence [23, 24]. In particular, liver recurrence is the most common recurrence pattern. Biomarkers that are closely associated with cancer progression and metastasis would allow early diagnosis and treatment. Therefore, the identification of novel prognostic and predictive molecular markers is essential in the planning of clinical strategies and development of new therapeutic approaches. At present, various molecular-targeted drugs have been developed and clinically applied [25–27]. However, CRC cannot be appropriately regulated, and the development of new biomarkers is therefore necessary.

PROK2, which was investigated in the present study, is a member of the prokineticin family and was isolated from skin secretions of the fire-bellied toad (*Bombina variegata*) [28]. It is closely related to endocrine gland-derived vascular endothelial growth factor (also called PROK1) and binds to two G protein-coupled receptors termed prokineticin receptor 1 (PK-R1) and prokineticin receptor 2 (PK-R2) [15, 29]. Members of the prokineticin family act as ligands of PK-R1 and PK-R2 and modulate several biological effects, including the stimulation of intracellular calcium levels, turnover of phosphoinositide, and induction of mitogen-activated protein kinase [30]. These signaling pathways shed some light on the actions of PROK2 on smooth muscle contractions and particularly on angiogenesis [29, 30]. Some previous studies have reported that PROK2 can promote the production of inflammatory cytokines, such as IL-1β and TNF-α, via PKR1 on macrophages [31–33]. The tumor microenvironment (niche) has recently been recognized as significant for the growth and progression of cancer cells [34]. Previous reports have demonstrated that inflammatory cytokines affect the microenvironment of existing cancer cells and facilitate the migration, invasion, and angiogenesis of cancer cells [35, 36]. The findings of our present and previous study [21] show that PROK2 expression is highly correlated with angiogenesis, tumor formation, and liver metastasis through *in vitro* and *in vivo* assays. This suggests that in CRC, the tumor itself organizes the surrounding environment via PROK2 expression, which plays a significant role within the tumor microenvironment. The significance of PROK2 in the tumor microenvironment has been highlighted by the present study, providing novel insight for the elucidation of the molecular mechanism underlying tumor invasion and metastasis for the development of a new therapy for CRC.

In this study, we detected positive PROK2 expression in primary CRC tissues in 50.9% (222/436) of patients with stage I–III CRC using immunohistochemistry. Consequently, we examined the impact of PROK2 expression on the clinical outcomes in patients with CRC. Our results revealed that positive PROK2 expression was closely associated with recurrence and inferior RFS. When individual CRC stages were analyzed, positive PROK2 expression was associated with a high incidence of liver recurrence and poor RFS duration in stage II and III CRC. Multivariate analysis showed that PROK2 expression in primary tumors was a significant independent predictor for RFS in patients with stage I–III CRC. In an *in vivo* animal model, we demonstrated that PROK2 expression was associated with enhanced liver metastasis. These results suggested that PROK2 is an extremely useful clinical biomarker for the prediction of prognosis of patients with CRC. To the best of our knowledge, this is the first study to demonstrate the utility of PROK2 as a prognostic biomarker for CRC.

Our findings have several clinical applications. First, PROK2 expression in primary tumors may be helpful to classify patients who require intensive surveillance following surgery. In spite of the recommendation of adjuvant chemotherapy for stage III patients, recurrence rates have been reported to exceed 30%–40% [37, 38]. Additionally, the recurrence rates are high for stage II CRC at 20%–30% [39, 40]. One of the main problems is that surveillance protocols after curative surgery in various countries require a careful balance between the assessment of cost-conscious programs and possibility of cancer recurrence, which prevents standardization. Additionally, there is a lack of consensus on the type of imaging and frequency of imaging/colonoscopy surveillance, and the decisions are frequently left to the discretion of the surgeon or oncologist. The surveillance regimens are generally applied across all stages of the disease, and this may limit the early detection of recurrence. It would therefore be extremely beneficial to have suitable indicators to predict a high risk of recurrence and suggest a more intensive follow-up. Therefore, biomarkers such as PROK2 may help by providing an indication of the need for aggressive surveillance. Second, the results of this study indicated that PROK2 could be a potential biomarker for the selection of stage II patients who may benefit from adjuvant chemotherapy. One of the possible reasons for the lack of a meaningful benefit of adjuvant therapy in stage II patients is the inability of the current prognostication system to identify those who are truly at high risk. Actually, there is no consensus regarding whether postoperative chemotherapy should be administrated to all the patients with stage II cancers [41]. Hence, the incorporation of PROK2 expression in primary tumors into current standard prognostication systems may allow better risk stratification, with a possible improvement in survival. In our study, PROK2 expression in primary tumors was clearly a significant independent predictor for RFS.

The present study had several limitations. First, participants were from a single institution, and the number of samples was relatively small. Second, this was a retrospective study. Third, the aim of this study was to identify novel molecular biomarkers with prognostic potential, regardless of their function; thus, we did not explore the biological mechanisms of PROK2 in tumor development. Therefore, future multicenter, prospective studies involving larger cohorts and functional experiments are needed to verify the robustness of our findings before clinical translation.

In conclusion, this study was the first to clinically demonstrate the importance of PROK2 as a prognostic biomarker in patients with CRC. Positive immunoreactivity of PROK2 was closely associated with liver recurrence. Additionally, PROK2 expression may serve as an independent predictor for poor outcomes in patients with CRC who are treated with surgical resection. Our findings may have important implications for the development of personalized medicine as PROK2 may allow the distinction of CRC patients who have a high risk of recurrence and require adjuvant chemotherapy from those who may benefit from curative surgery alone. We believe that our results might enhance the development of novel therapeutic strategies for CRC.

## MATERIALS AND METHODS

### Patients and samples

Colorectal cancer and adjacent normal tissues were obtained from surgically resected specimens of 436 patients with stage I–III CRC who underwent curative surgery at the First Department of Surgery, University of Fukui, Japan, between 1992 and 2006. All the samples were fixed in 12% paraformaldehyde (pH 6.7) for 24 h and embedded in paraffin. Clinicopathological data were obtained from the histopathological reports and clinical records of the patients. TNM staging was assessed according to the American Joint Committee on Cancer Staging Manual (seventh edition) [42]. Tumors were classified as stage I in 92 (21.1%) specimens, stage II in 168 (38.5%), and stage III in 176 (40.4%). As histopathological findings differed within the same tumors, the diagnosis was evaluated in conformity to the dominant type by two pathologists.

The inclusion criteria for the study were as follows: specific histopathological findings confirming primary CRC, resection of CRC with extended (D2 or D3) lymphadenectomy [43], histological curative resection (stages I–III), Eastern Cooperative Oncology Group performance status (PS: 0, 1, or 2), no preoperative chemotherapy or radiotherapy, tegafur/uracil-based postoperative chemotherapy in patients with stage III disease, and no chemotherapy in patients with stage I or II disease after surgical resection. All the patients were followed up for recurrence every 3 or 6 months for 5 years, and they were submitted to chest radiography, computed tomography, and colonoscopy according to the guidelines of the Japanese Society for Cancer of the Colon and Rectum. The median follow-up term was 63.5 months (range, 12.0–219.0 months). RFS was defined as the duration from the date of primary resection to first cancer recurrence, death from the disease, or last follow-up. The present study was approved by the Ethics Committee of the University of Fukui, Japan. All the patients provided written informed consent before inclusion in this study.

### Immunohistochemical staining

Immunohistochemical staining for PROK2 was performed according to a standard avidin–biotin complex method. All the specimens were embedded in paraffin and sliced into 4-μm-thick sections. Subsequently, the sections were deparaffinized in xylene and rehydrated through graded ethanol baths (5 min in each ethanol concentration). Endogenous activity was blocked by incubation with 1% hydrogen peroxidase in methanol for 30 min. After blocking nonspecific binding sites using dilution of skim milk powder for 30 min, the hydrated sections were incubated with anti-PROK2 antibody (Novus Biochemicals, Littleton, CO, USA) in a humidity chamber at 4°C overnight. After washing the sections with phosphate buffered saline (PBS), the signal of PROK2 protein was developed using the ChemMate method (Dako, Glostrup, Denmark). Finally, the sections were counterstained with hematoxylin and examined using light microscopy. PROK2 expression was considered positive when more than 30% of the tumor cells showed positive staining on analysis using Image J software (http://rsb.info.nih.gov/ij/) [44].

### Cell lines and treatments

The human CRC cell lines LoVo, SW480, DLD-1, and HCT116 were obtained from the American Type Culture Collection (ATCC). All the cell lines were cultured in RPMI 1640 medium (Sigma, St. Louis, MO, USA) containing 10% fetal bovine serum (FBS), 100 U/mL penicillin, and 100 μg/mL streptomycin at 37°C in a 5% CO_2_ atmosphere. The medium was replaced with fresh medium every 2 or 3 days, and each cell line was passaged at 70% confluence in a 100-mm culture plate using 0.25% trypsin-EDTA (Gibco, Gaithersburg, MD, USA).

### RNA extraction and RT-PCR

Total RNA was purified from CRC cell lines with ISOGEN II (Wako, Tokyo, Japan). Complementary DNA (cDNA) was synthesized from total RNA using the Prime Script RT reagent kit (Takara, Otsu, Japan) and the template for reverse transcription. Gene expression of PROK2 was analyzed using PCR. The primer sequences for PROK2 (GenBank accession no. NM_021935) gene-coding regions were as follows: 5′ primer, PROK2-AX: 5′-GGGGATCCATGAGGAGCCTGTGCTGCGCCCCA-3′; 3′ primer, PROK2-BX: 5′-GGGAATTCCTTTTGGGCTAAACAAATAAATCG-3′ [21]. GAPDH was used as an internal PCR control. The amplification conditions were as follows: 1) denaturation at 95°C for 1 min; 2) annealing at 55°C for 1.5 min; and 3) extension at 72°C for 2.5 min. Thirty-five cycles were performed using a thermal cycler (PTC-100, Programmable Thermal Controller; MJ Research Inc. Watertown, MA, USA). The result of amplification was analyzed using 2% agarose gel electrophoresis. Sequencing was performed on PCR products for clarifying the bands in RT-PCR analysis. Finally, the gels were stained with ethidium bromide to confirm the band of PROK2 mRNA. The reactive expression level of the target gene was standardized to that of the GAPDH internal control. All experiments were repeated three times to confirm reproducibility.

### Plasmid construction

The pCMV6-Ac-PROK2 vector was purchased from OriGene (Rockville, MD, USA), whereas the pCMV6-Ac-empty vector was purchased from Invitrogen (Carlsbad, CA, USA). PROK2 cDNA was amplified using the following primers: 5′ primer, encompassed position, PROK2-CX: 5′-GGGGATCCGGTACCGAGGAGATCTGCCG-3′; 3′ primer, encompassed position, PROK2-DX: 5′-GGGAATTCGGCCGTTTAAACTCTTTCTTC-3′. Thermal cycler conditions were as follows: denaturation, 95°C for 1 min; annealing, 50°C for 1.5 min; and extension, 72°C for 2.5 min. Thirty-five cycles were performed. The BamHI and EcoRI site-tagged full-length PROK2-GFP fragments were amplified and cloned into a mammalian expression vector between the BamHI and EcoRI sites [21]. The plasmid structures were identified using DNA sequencing. We established a constitutive expression vector of human PROK2-GFP (cCMV6-Ac-PROK2-GFP).

### Transfection

To overexpress PROK2, HCT116 and DLD-1 were transfected with pCMV6-Ac-GFP-PROK2 or pCMV6-Ac-empty vector (as control). The cells were transfected using suitable amounts of plasmid DNA with Lipofectamine 3000 reagent (Invitrogen) and Opti-MEM (Invitrogen) according to the manufacturer’s protocols. The cells were allowed to grow for 48 h following transfection. The cells were then isolated according to neomycin resistance by treatment with 600 μg/mL of G418 sulfate (Promega, Madison, WI, USA) for 3 weeks. The cells expressing PROK2 were validated using focal laser microscopy, RT-PCR, and western blot analysis.

### Western blot analysis

HCT116 and DLD-1 cells were placed into 6-well plates (5 × 10^4^ cells/well) with RPMI containing 10% FBS and incubated at 37°C in 5% CO_2_. Total cell protein was extracted using RIPA lysis buffer supplemented with protease inhibitors (phenylmethylsulfonyl fluoride, leupeptin, and sodium orthovanadate). Denatured proteins were separated by SDS-PAGE using 15% SuperSep Gel (Wako, Japan). The resolved proteins were transferred to a polyvinylidene difluoride (PVDF) membrane using the wet-transfer apparatus (Bio-Rad, Hercules, CA, USA) [45]. Following electrophoretic transfer, the protein bands were blocked in 5% skim milk overnight at 4°C and incubated with anti-PROK2 antibody (Novus Biochemicals). The blots were washed thrice in T-TBS and incubated with horseradish peroxidase (HRP)-tagged goat anti-rabbit IgG secondary antibodies for 1 h at room temperature. Immunoreactivity was visualized using enhanced chemiluminescence according to the manufacturer’s instructions (Amersham, Piscataway, NJ, USA). The experiment was performed in triplicate.

### *In vivo* liver metastasis model

A liver metastasis assay was performed to assess the effects of PROK2 on CRC metastasis. Male SHO nude mice matured up to six weeks (Charles River, Wilmington, MA, USA) were anesthetized, and the abdomen was prepared for sterile surgery. A left abdominal flank dissection was performed, and the spleen was identified. Using a 27-gage needle, 50 μL (1.0 × 10^6^ cells/50 μL in PBS) of PROK2-transfected DLD-1/HCT116 or empty vector-transfected DLD-1/HCT116 (as control) was orthotopically injected under the splenic capsule (n = 20 mice per cell line). Four weeks after splenic infection, the mice were sacrificed and the abdominal organs and thorax were evaluated for macroscopic and microscopic metastases. All mouse livers were fixed in formalin and cut into 4-μm-thick sections for histopathological analysis. Metastatic nodules were counted using microscopy in a double-blinded manner. The number of metastatic liver clones in each cell line was compared between the PROK2 transfected and control mice groups. The study was performed in accordance with the standard procedure approved by the University of Fukui.

### Statistical analysis

The correlations between PROK2 expression and various clinicopathological characteristics were assessed using cross tabulation, and statistical evaluations were performed using the chi-square or Mann–Whitney *U* test. RFS curves were calculated using the Kaplan–Meier method, and the differences between the groups were assessed by the log-rank test. Cox regression analyses were performed to evaluate the independent predictive factors for RFS. HRs and 95% CIs were subsequently calculated. A p-value of <0.05 was considered statistically significant. All statistical analyses were performed using SPSS software (IBM SPSS Statistics, IBM Corp., Armonk, NY, USA).

## Abbreviations

ATCC: American Type Culture Collection
CRC: Colorectal cancer
GAPDH: Glyceraldehyde-3-phosphate dehydrogenase
PBS: Phosphate buffered saline
PVDF: Polyvinylidene difluoride
RFS: Recurrence-free survival
RT-PCR: Reverse transcription-polymerase chain reaction
TNM: Tumor-node-metastasis
